# Multicentre study of prepectoral breast reconstruction using acellular dermal matrix

**DOI:** 10.1002/bjs5.50236

**Published:** 2019-12-19

**Authors:** M. Chandarana, S. Harries, on behalf of the National Braxon Audit Study Group, A. Tenovici, C. Mortimer, D. Clarke, D. Banerjee, D. Thekkinkattil, D. Predolac, D. Ferguson, E. Vaughan‐Williams, G. Osborne, G. Mitchell, I. Azmy, L. Jones, N. Roche, P. Bhaskar, R. Achuthan, R. Rathinaezhil, R. Parmeshwar, S. Narayanan, S. Seetharam, S. Marla, S. Soumian, T. Sircar

**Affiliations:** ^1^ Department of General Surgery Forth Valley Royal Hospital Larbert UK; ^2^ Department of Breast Surgery Warwick Hospital, South Warwickshire NHS Foundation Trust Warwick UK

## Abstract

**Background:**

Single‐stage reconstruction is used widely after mastectomy. Prepectoral implant placement is a relatively new technique. This multicentre audit examined surgical outcomes following prepectoral reconstruction using acellular dermal matrix (ADM).

**Methods:**

All patients who had a mastectomy with prepectoral breast reconstruction and ADM in the participating centres between January 2015 and December 2017 were included. Demographic and treatment details, and short‐ and long‐term operative outcomes were recorded. Factors affecting complications and implant loss were analysed: age, BMI, smoking status, diabetes, vascular disease, laterality of surgery, previous ipsilateral breast surgery or radiotherapy, indication for surgery (invasive *versus in situ* carcinoma, or risk reduction), type of mastectomy, axillary clearance, breast volume, implant volume, and neoadjuvant and adjuvant chemotherapy.

**Results:**

A total of 406 reconstructions were performed across 18 centres. Median follow‐up was 9·65 months. Median hospital stay was 1 day. The 90‐day unplanned readmission rate was 15·7 per cent, and the return‐to‐theatre rate 16·7 per cent. Some 15·3 per cent of patients had a major complication, with a 90‐day implant loss rate of 4·9 per cent. A further six patients had delayed implant loss. In multivariable analysis, no factor was significantly associated with complications or implant loss.

**Conclusion:**

Prepectoral breast reconstruction with ADM has satisfactory surgical outcomes. The duration of follow‐up needs to be extended to examine outcomes in patients who received adjuvant radiotherapy.

## Graphical abstract

A UK multicentre audit of direct‐to‐implant prepectoral breast reconstructions using acellular dermal matrix was conducted for all patients operated on from January 2015 to December 2017 across 18 centres. In a median follow‐up of 9·7 months, the major complication rate was 15·3 per cent and the 90‐day implant removal rate 4·9 per cent. Outcomes were satisfactory and comparable to national data reported in studies on subpectoral reconstruction.

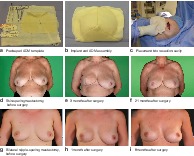

Longer follow‐up needed to confirm good early outcomes

## Abstract (es)

**Antecedentes:**

La reconstrucción en un tiempo es una técnica muy utilizada tras la mastectomía. La colocación de un implante prepectoral es una técnica relativamente novedosa. Esta auditoría multicéntrica analizó los resultados quirúrgicos después de la reconstrucción prepectoral utilizando una matriz dérmica acelular (*acellular dermal matrix*, ADM).

**Métodos:**

Se incluyeron todas las pacientes en las que se efectuó una reconstrucción mamaria prepectoral y ADM tras la mastectomía en los centros participantes desde enero de 2015 hasta diciembre de 2017. Se analizaron los datos demográficos, detalles del tratamiento y los resultados operatorios a corto y a largo plazo. Se evaluaron los factores relacionados con las complicaciones y la pérdida del implante, incluyendo la edad, el índice de masa corporal, el tabaquismo, la diabetes, la enfermedad vascular, la lateralidad de la cirugía, la cirugía mamaria ipsilateral previa o la radioterapia previa, la indicación de cirugía (carcinoma invasivo *versus* carcinoma in situ o reducción del riesgo), el tipo de mastectomía, el vaciamiento axilar, el volumen mamario, el volumen del implante y la quimioterapia neoadyuvante y adyuvante.

**Resultados:**

Se incluyeron 406 reconstrucciones efectuadas en 18 centros. La mediana de seguimiento fue de 9,65 meses. La mediana de la estancia hospitalaria fue de un día. La tasa de reingreso no planificado a los 90 días fue de 15,74% y la tasa de reoperaciones fue del 16,66%. Alrededor del 15% de las pacientes presentaron una complicación mayor, con una tasa de pérdida de implante del 4,93% a los 90 días. En otras 6 pacientes se retiró el implante con posterioridad. En el análisis multivariable no se identificó ningún factor que se asociara significativamente con la tasa de complicaciones o la pérdida del implante.

**Conclusión:**

La reconstrucción mamaria prepectoral con ADM tiene resultados quirúrgicos satisfactorios. Se debe ampliar el periodo de seguimiento para analizar los resultados en pacientes tratadas con radioterapia adyuvante.

## Introduction

Women undergoing mastectomy for breast cancer or risk reduction should be offered an immediate breast reconstruction if deemed suitable^1^. The 2011 UK National Mastectomy and Breast Reconstruction Audit^2^ showed that about 40 per cent of women diagnosed with preinvasive or invasive breast cancer undergo mastectomy. In addition, there is increasing demand from women requesting risk reduction surgery. The past decade has seen a steady rise in implant and expander‐based immediate breast reconstruction, from about 30 per cent in 2007 to 54 per cent in 2013^3^. There has been a gradual shift from a two‐stage approach to a single‐stage direct‐to‐implant procedure, so that one‐stage prosthetic reconstruction has become the standard technique of breast reconstruction in many centres^4^. Subpectoral implant placement has been the conventional method, with complete coverage of the implant using pectoralis major muscle above and acellular dermal matrix (ADM) in the lower and outer aspect. Complete implant coverage provides a larger pocket for implant placement and better control of the inframammary fold. However, animation deformity and postoperative pain related to detachment of pectoralis major muscle remain concerns^5^, ^6^, ^7^, ^8^.

Prepectoral implant placement is a relatively new technique that avoids detachment of the pectoralis major muscle. The implant is placed in the prepectoral pocket created after skin‐ or nipple‐sparing mastectomy, and is usually covered by a biological or synthetic mesh. The main advantages over the subpectoral technique are claimed to be avoidance of disruption of pectoralis major muscle, less postoperative pain, no animation deformity and less capsular contracture^5^, ^9^, ^10^, ^11^, ^12^. Some evidence^11^, ^13^, ^14^, ^15^ suggests comparable, or even superior, surgical, aesthetic and cost‐effective outcomes for the prepectoral technique compared with subpectoral implant placement. The use of ADM for direct‐to‐implant prepectoral reconstructions has been described in a number of studies^16^, ^17^, ^18^, ^19^, ^20^, ^21^, ^22^, ^23^, ^24^, ^25^, ^26^ reporting short‐ and long‐term outcomes. Braxon® (MBP Biologics, Neustadt‐Glewe, Germany; licence‐holder DECO med, Marcon, Venezia, Italy) is a novel biological mesh used for prepectoral implant‐based immediate breast reconstruction. It is a porcine dermis‐derived ADM of 0·6 mm in thickness, available as a preshaped template to be wrapped around the implant *ex vivo*. It allows complete coverage of the implant in the prepectoral pocket created after skin‐ or nipple‐sparing mastectomy. Two recent multicentre European studies^13^, ^18^ reported acceptable operative outcomes, comparable to subpectoral reconstruction.

The present study reports on surgical outcomes from a multicentre audit conducted in the UK on prepectoral direct‐to‐implant reconstruction using ADM, and factors affecting complication rates and implant loss.

## Methods

The audit was initiated in August 2017. All centres across the UK performing prepectoral breast reconstructions using Braxon® ADM were invited to participate. Centres were requested to submit data on all consecutive patients undergoing skin‐sparing, nipple‐sparing or skin‐reducing mastectomy with prepectoral implant‐based reconstruction using Braxon® from January 2015 to December 2017. A National Braxon Audit Study Group included all surgeons who contributed data. Association of Breast Surgery (ABS) and British Association of Plastic, Reconstructive and Aesthetic Surgeons (BAPRAS) guidelines^1^ were followed by participating centres for patient selection for prepectoral reconstruction. All patients operated on in the given period who had a minimum follow‐up of 3 months were included. Patients who had a reconstruction using subpectoral implant placement or tissue expanders were excluded. Centres that could not submit data for patients treated before March 2018 were excluded.

Data were collected on patient demographics, treatment details, tumour characteristics and postoperative outcomes for a minimum of 90 days after surgery. Implant losses occurring after 90 days were recorded. Centres used intraoperative and postoperative antibiotics according to local protocols. Operative technique of skin‐ or nipple‐sparing mastectomy, perioperative management and drain insertion at the surgical site reflected institutional protocols or surgeon preference.

Outcomes studied were unplanned readmissions, return to theatre, postoperative complications and rate of implant loss. Specific complications recorded were seroma, skin redness or red breast syndrome, skin necrosis, infection, wound dehiscence, postoperative haematoma and capsular contracture. Redness of the wound or breast with no documented evidence of infection was classified as redness or red breast syndrome. Redness accompanied by any systemic sign of infection, positive bacteriology culture from the surgical site or blood, or a diagnosis of infection according to the treating surgeon's decision was classified as wound infection. Complications were categorized as major or minor based on the Clavien–Dindo grading system^27^. All complications of grade III or higher were categorized as major complications. Operative intervention with removal of implant was considered as implant loss. Return to theatre with exchange of implant to another implant or an expander was not considered as implant loss, but as a major complication. Implant loss or complications occurring up to 90 days after surgery were categorized as early implant loss or complications respectively. Implant loss recorded after 90 days of reconstruction was considered as delayed implant loss. Age, BMI, smoking status, diabetes, vascular disease, laterality of surgery, previous ipsilateral breast surgery or radiotherapy, indication for surgery (invasive *versus in situ* carcinoma, or risk reduction), type of mastectomy, axillary clearance, breast volume, implant volume, and neoadjuvant and adjuvant chemotherapy were the factors studied for their impact on major complications and implant loss rates. Adjuvant radiotherapy was not considered for its impact on early complications or implant loss as it was administered after completion of adjuvant chemotherapy, more than 90 days after the primary operation.

### Statistical analysis

Data are presented as mean(s.d.) or median (range) values, or as numbers of patients or procedures with percentages as appropriate. The χ^2^ test was used for univariable analysis to calculate significance of contributing factors. *P* < 0·050 was taken as significant. Factors found to be significant in univariable analysis were included in the multivariable model, and statistical significance was determined by logistic regression. IBM SPSS® 24.0 (IBM, Armonk, New York, USA) was used for statistical calculations and analysis.

## Results

**Figure 1:**
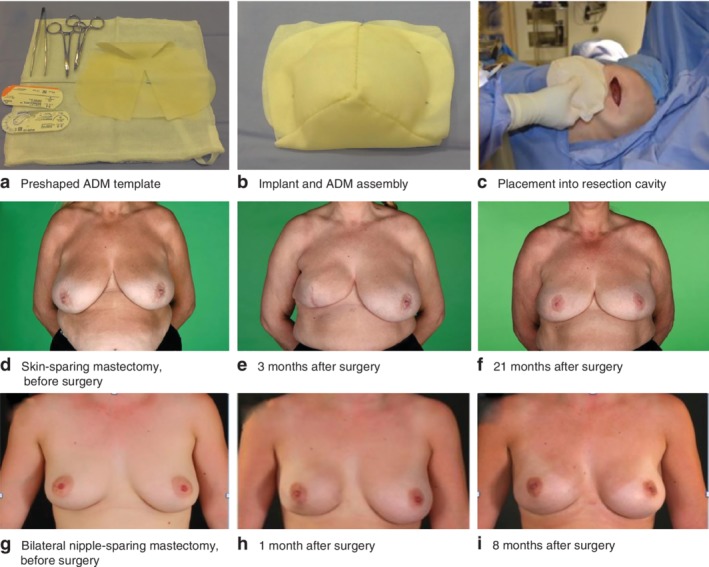
Braxon® device assembly and surgical outcomes **a** Acellular dermal matrix (ADM) available as a preshaped template; **b** implant and ADM assembly; **c** placement of implant and ADM into the resection cavity. **d** Skin‐sparing mastectomy, before surgery; **e** 3 months after surgery; **f** 21 months after surgery. **g** Bilateral nipple‐sparing mastectomy, before surgery; **h** 1 month after surgery; **i** 8 months after surgery.

A total of 324 women underwent 406 reconstructions across 18 centres in the UK (*Table* *S1*, supporting information). Patient characteristics and treatment details are shown in *Table* 1. Their median age was 49 years and median BMI was 25 kg/m^2^. Bilateral procedures were performed in 82 women. Half of the procedures were performed for invasive cancer, the rest for preinvasive cancer or risk reduction. Apart from two women who had a secondary reconstruction, all patients had immediate prepectoral breast reconstruction following mastectomy (*Fig*. 1). The median duration of hospital stay was 1 day. Median duration of follow‐up for the cohort was 9·7 (range 3–35; mean 11) months; 168 women were followed for more than 12 months.

**Table 1:** Patient characteristics and treatment details

	No. of patients or procedures*
**Total no. of patients**	324
**Total no. of procedures**	406
**Age (years)** †	49 (20–82)
**BMI (kg/m** ^**2**^ **)** †	25 (18–43)
**Laterality**	
Unilateral	242 (74·7)
Bilateral	82 (25·3)
**Smoker**	15 (4·6)
**Diabetes**	7 (2·2)
**Vascular disease**	6 (1·9)
**Previous breast surgery**	69 (17·0)
**Previous breast radiotherapy**	15 (3·7)
**Indication for surgery**	
Invasive disease	204 (50·2)
*In situ* carcinoma	90 (22·2)
Risk reduction/revision	105 (25·9)
Missing	7 (1·7)
**Type of mastectomy**	
Skin‐sparing	224 (55·2)
Nipple‐sparing	143 (35·2)
Skin‐reducing	37 (9·1)
Missing	2 (0·5)
**Management of axilla**	
Sentinel node biopsy	227 (55·9)
Axillary nodal clearance	54 (13·3)
None	104 (25·6)
Missing	21 (5·2)
**Length of hospital stay (days)** †	1 (0–10)
**Weight of breast (g)** †	376 (64–3900)
**Implant volume (ml)** †	370 (105–685)
**Neoadjuvant chemotherapy**	49 (15·1)
**Adjuvant chemotherapy**	57 (17·6)
**Adjuvant radiotherapy**	62 (15·3)

* With percentages in parentheses unless indicated otherwise;

† values are median (range).

Of the 406 procedures, 116 resulted in complications, an overall complication rate of 28·6 per cent (*Table* 2): 62 major (15·3 per cent) and 54 minor (13·3 per cent) complications. For management of complications, 51 women (15·7 per cent of the cohort) needed an unplanned readmission and 54 (16·7 per cent) had a surgical exploration within 90 days of the primary operation. Of these, 44 women (13·6 per cent of the cohort) had a surgical exploration for implant‐related complications. Some 26 implants were removed, giving an overall implant loss rate of 6·4 per cent. Of these, 20 implants were removed within 90 days of the primary surgery (*Table* *S2*, supporting information), with an implant loss rate at this time of 4·9 per cent. Six women had a delayed implant loss, more than 3 months after the reconstruction. No patient with delayed implant loss had received adjuvant radiotherapy.

**Table 2:** Complications

	No. of complications (*n* = 406)
Seroma	29 (7·1)
Skin necrosis	21 (5·2)
Red breast syndrome	16 (3·9)
Infection	13 (3·2)
Haematoma	10 (2·5)
Wound dehiscence	8 (2·0)
Pain	1 (0·2)
Capsular contracture	1 (0·2)
Other	3 (0·7)
Missing	14 (3·4)
Total	116 (28·6)

Of the factors studied to determine any association with early major complications or implant loss, major complications were significantly higher in univariable analysis in patients with a high BMI (*P* = 0·002), greater excised breast volume (*P* = 0·001), larger implant size (*P* = 0·005) and axillary nodal clearance (*P* = 0·002). Similarly, patients with a higher BMI (*P* = 0·009), greater excised breast volume (*P* = 0·017) and larger implant size (*P* = 0·004) had a significantly higher implant loss. None of the factors had a significant impact on major complications or implant loss in multivariable analysis (*Table* 3).

**Table 3:** Univariable and multivariable analysis

	Major complications		Implant loss	
	Univariable *P* *	Multivariable *P* †	Univariable *P* *	Multivariable *P* †
**Age**	0·116		0·958	
**BMI**	0·002	0·135	0·009	0·347
**Smoker**	0·929		0·26	
**Diabetes**	1·000		0·363	
**Vascular disease**	1·000		0·320	
**Bilateral reconstruction**	0·084		0·119	
**Previous breast surgery**	0·351		0·594	
**Previous breast radiotherapy**	1·000		0·612	
**Invasive breast carcinoma**	0·143		0·905	
**Type of mastectomy**				
Skin‐sparing	0·729		0·154	
Nipple‐sparing				
Skin‐reducing				
**Axillary nodal clearance**	0·002	0·063	0·072	
**Breast weight**	0·001	0·322	0·017	0·361
**Implant volume**	0·005	0·218	0·004	0·070
**Neoadjuvant chemotherapy**	0·778		0·749	
**Adjuvant chemotherapy**	0·209		1·000	

* χ^2^ test;

† logistic regression.

## Discussion

The introduction of ADM in implant‐based reconstruction has made prepectoral implant placement and complete implant coverage possible, with excellent surgical and aesthetic outcomes. This multicentre audit has demonstrated good short‐ and long‐term surgical outcomes, with an implant loss rate of 6·4 per cent after 9 months, a 90‐day major complication rate of 15·3 per cent and implant‐related return‐to‐theatre rate of 13·6 per cent, with a resultant 90‐day implant loss rate of only 4·9 per cent.

Braxon® is the only ADM currently available as a preshaped template that allows complete implant coverage *ex vivo*, facilitating direct‐to‐implant reconstruction of implant sizes of up to 520 ml. Other case series using this product indicated satisfactory short‐term clinical and aesthetic outcomes. The implant loss rate was 12 per cent with a seroma rate of 8 per cent, using a previously manufactured thicker (0·9 mm) version^16^. A long‐term outcome study^17^ of ten patients, with a median follow‐up of 49·2 months, reported no capsular contracture rates and minimal implant rippling in two patients. A study^21^ that included 51 reconstructions found skin necrosis, seroma and implant loss rates of 4 per cent each. Two multicentre studies^18^, ^20^ have reported on short‐term outcomes of prepectoral reconstruction using prepectoral implant and ADM. The first^18^ reported on 100 reconstructions across nine centres in Europe with an implant loss rate of 2 per cent and a perioperative complication rate of 11 per cent. The other study^20^ included 78 reconstructions reported from three centres in the UK; the implant loss rate was 10·2 per cent and the complication rate about 20 per cent. These results were comparable to national data on implant‐based reconstructions in the UK^2^, ^28^, ^29^.

Information on outcomes from other ADMs and synthetic meshes for prepectoral implant‐based immediate breast reconstruction is limited^22^, ^23^, ^25^, ^26^. A recent study^26^ included 166 reconstructions of which three‐quarters had complete implant coverage using one or two sheets of ADM, whereas others had a dermal sling partially covering the implant. About one‐third of the patients had a subpectoral to prepectoral conversion, about 9 per cent had an expander placement, and around 70 per cent had risk‐reducing surgery, compared with approximately 25 per cent in the present audit. The overall complication rate was 11·5 per cent and implant loss rate 3 per cent.

There have been two national audits from the UK describing breast reconstruction outcomes^2^, ^28^. The first audit^2^ covered breast reconstructions performed from January 2008 to March 2009 with an implant‐based reconstruction cohort of more than 3000 patients. The overall complication rate in this group was 14·7 per cent, with a return‐to‐theatre rate of 4·6 per cent and an implant loss rate of 9 per cent, although all were placed in a subpectoral position^2^. The more recent implant‐based breast reconstruction evaluation (iBRA) study^28^ included 2108 patients operated on for implant‐based reconstruction between February 2014 and June 2016, but only 42 (2·0 per cent) who had a prepectoral reconstruction. Overall readmission and return‐to‐theatre rates were about 18 per cent, and the 3‐month implant loss rate was 9 per cent. Outcomes in the prepectoral subgroup were analysed separately and considered comparable to the overall results^28^. The 90‐day implant loss rate in the present study was only 4·9 per cent, with an infection rate of 3·2 per cent, both of which are considerably lower than results reported in the national audits (*Table* 4).

**Table 4:** Comparison of present results with recommended quality criteria, National Mastectomy and Breast Reconstruction Audit and implant‐based Breast Reconstruction Evaluation results

	ABS/BAPRAS recommendation (%)	NMBRA (%)	iBRA (%)	iBRA prepectoral group (%)	National Braxon® Audit (%)
Unplanned readmission	< 5	16	18	24	15·7
Return to theatre for local complications	< 5	4·6	18	21	16·7
90‐day infection rate	< 10	25	25	26	3·2
90‐day implant loss rate	< 5	9	9	7	5·2

ABS, Association of Breast Surgery; BAPRAS, British Association of Plastic, Reconstructive and Aesthetic Surgeons; NMBRA, National Mastectomy and Breast Reconstruction Audit; iBRA, implant‐based Breast Reconstruction evAluation.

Selection criteria and surgical procedures were based on ABS and BAPRAS guidelines^1^. In the selected cohort, 42 women had a BMI above 30 kg/m^2^ and were counselled regarding an increased risk of complications. Patients with a higher BMI, greater excision volume and larger implant size did have significantly higher complication rates and implant loss in univariable analysis, although no factor remained significant in multivariable analysis.

This study has limitations. It is a retrospective multicentre study and will have the disadvantages of a retrospective analysis^30^. There is likely to have been variation in patient selection, surgical techniques and perioperative management among the participating centres. Aesthetic and patient‐reported outcomes were not reported. Postoperative pain scores were available for only a minority of patients and could not be included as an outcome measure. The median duration of follow‐up of 9·7 months was relatively short, and there have been reports^31^, ^32^ of higher complication rates and implant losses with longer follow‐up intervals in patients with subpectoral reconstructions. In this series, only six implants needed removal beyond 90 days of follow‐up, but much longer follow‐up is necessary to identify late outcomes, particularly in women receiving adjuvant radiotherapy.

## Collaborators

The following are members of the National Braxon Audit Study Group: A. Tenovici, C. Mortimer, D. Clarke, D. Banerjee, D. Thekkinkattil, D. Predolac, D. Ferguson, E. Vaughan‐Williams, G. Osborne, G. Mitchell, I. Azmy, L. Jones, N. Roche, P. Bhaskar, R. Achuthan, R. Rathinaezhil, R. Parmeshwar, S. Narayanan, S. Seetharam, S. Marla, S. Soumian, T. Sircar.

## Supporting information


**Table S1.** List of centres and number of procedures
**Table S2.** Details of patients with implant loss
