# Supplementary material for: Multicentre study of prepectoral breast reconstruction using acellular dermal matrix
Source: BJS Open. 2019 Dec 19;4(1):71–7. doi: 10.1002/bjs5.50236 (PMC6996627; doi:10.1002/bjs5.50236)
Supplement: Supplementary file 1 — Table S1. List of centres and number of procedures Table S2. Details of patients with implant loss [file BJS5-4-71-s001.docx]

**BJS5_50236**

**Multicentre study of prepectoral breast reconstruction using acellular dermal matrix**

**M. Chandarana and S. Harries**

**Table S1** List of centres and number of procedures

| Centre number | Number of procedures |
| --- | --- |
| 1 | 19 |
| 2 | 14 |
| 3 | 21 |
| 4 | 11 |
| 5 | 101 |
| 6 | 11 |
| 7 | 13 |
| 8 | 5 |
| 9 | 12 |
| 10 | 25 |
| 11 | 14 |
| 12 | 25 |
| 13 | 53 |
| 14 | 14 |
| 15 | 11 |
| 16 | 11 |
| 17 | 26 |
| 18 | 20 |
| Total | 406 |

**Table S2** Details of patients with implant loss

| Sr. No. | Breast weight | Implant volume | Adjuvant chemotherapy | Adjuvant RT | Complication | Date of second surgery | Time period |
| --- | --- | --- | --- | --- | --- | --- | --- |
| 1 | 458, 248 | 360 | No | No | Seroma | 01.07.2015 | Delayed |
| 2 | NA | 440 | No | No | Infection | 29.02.2016 | Delayed |
| 3 | 440 | 405 | No | No | Seroma | 02.06.2016 | Delayed |
| 4 | NA | 535 | No | No | Infection | 13.05.2016 | Early |
| 5 | 235 | 215 | No | No | Wound dehiscence | 25.06.2016 | Early |
| 6 | 461 | 375 | No | No | Infection |  | NA |
| 7 | 546 | 515 | Yes | Yes | Skin necrosis | 21.10.2016 | Early |
| 8 | 580, 580 | 520, 520 | No | No | Infection | 26.10.2016 | Early |
| 9 | 1410, 1215 | 535, 535 | No | No | NA | 25.11.2016 | Early |
| 10 | 245 | 425 | No | No | Infection | 22.12.2016 | Early |
| 11 | 3900, 3550 | 535, 535 | No | No | NA | 29.12.2016 | Early |
| 12 | 292 | 330 | Yes | No | Patient choice* | 07.12.2017 | Delayed |
| 13 | 520 | 445 | No | No | Infection | 04.02.2017 | Early |
| 14 | NA | 440 | No | No | Wound dehiscence | 27.02.2017 | Early |
| 15 | NA | 265 | Yes | No | Wound dehiscence | 29.03.2017 | Early |
| 16 | 186 | 420 | No | No | NA | 22.05.2017 | Early |
| 17 | NA | 310 | No | No | Infection | 08.11.2017 | Delayed |
| 18 | 394 | 450 | No | No | Wound dehiscence | 19.09.2017 | Early |
| 19 | 475 | 485 | No | No | Infection | 06.12.2017 | Early |
| 20 | 1032 | 595 | No | No | Skin necrosis | 01.11.2017 | Early |

*The patient did not have any complications from the reconstruction but demanded removal of the implant after about 10 months of reconstruction.

RT – Radiotherapy, NACT – Neoadjuvant chemotherapy, NSM – Nipple-sparing mastectomy, SSM – Skin-sparing mastectomy, SNB – Sentinel node biopsy, ANC – Axillary nodal clearance, NA – Not available
